# Manipulation of Pre-Target Activity on the Right Frontal Eye Field Enhances Conscious Visual Perception in Humans

**DOI:** 10.1371/journal.pone.0036232

**Published:** 2012-05-15

**Authors:** Lorena Chanes, Ana B. Chica, Romain Quentin, Antoni Valero-Cabré

**Affiliations:** 1 Groupe de Dynamiques Cérébrales, Plasticité et Rééducation, Equipe Cognition, Neuro-Imagerie et Maladies du Cerveau, CNRS UMR 7225-INSERM UMRS 975-UPMC (Paris VI), Centre de Recherche de l’Institut du Cerveau et la Moelle (CRICM), Paris, France; 2 Department of Experimental Psychology, University of Granada, Granada, Spain; 3 Laboratory for Cerebral Dynamics Plasticity and Rehabilitation, Department of Anatomy and Neurobiology, Boston University School of Medicine, Boston University, Boston, Massachusetts, United States of America; 4 Cognitive Neuroscience and Information Technology Research Program, Open University of Catalonia (UOC), Barcelona, Spain; University of California, Davis, United States of America

## Abstract

The right Frontal Eye Field (FEF) is a region of the human brain, which has been consistently involved in visuo-spatial attention and access to consciousness. Nonetheless, the extent of this cortical site’s ability to influence specific aspects of visual performance remains debated. We hereby manipulated pre-target activity on the right FEF and explored its influence on the detection and categorization of low-contrast near-threshold visual stimuli. Our data show that pre-target frontal neurostimulation has the potential when used alone to induce enhancements of conscious visual detection. More interestingly, when FEF stimulation was combined with visuo-spatial cues, improvements remained present only for trials in which the cue correctly predicted the location of the subsequent target. Our data provide evidence for the causal role of the right FEF pre-target activity in the modulation of human conscious vision and reveal the dependence of such neurostimulatory effects on the state of activity set up by cue validity in the dorsal attentional orienting network.

## Introduction

Since the pioneering studies by Posner and collaborators [1], the ability of visuo-spatial attentional orienting to influence visual performance has been widely demonstrated. More recent work has specifically reported enhancements in several aspects of visual perception such as spatial resolution, contrast sensitivity and orientation discrimination in those regions of the visual field where attention is willfully focused or involuntarily captured [2], [3], [4]. Such facilitatory phenomena are thought to be mediated by the ability of long-range connectivity from non-visual regions to reduce background noise, sharpen the tuning, boost the gain, or reduce the variance in firing activity of neuronal populations located within primary visual areas [5], [6].

Solid neuroimaging evidence of the human brain has so far helped identify a dorsal network involved in visuo-spatial attentional orienting, with the participation among others, of key cortical sites such as the right Intraparietal Sulcus (IPS) and the Frontal Eye Fields (FEF) [7]. This dorsal system would be supplemented by a ventral network, which would act as a “circuit breaker”, allowing the re-orientation of attention to unexpected and task-relevant events [7], [8]. Interestingly, some of these sites appear to co-localize with the nodes of a distributed long-range connectivity network, which, according to theoretical models and neuroimaging data, might play an essential role in access to consciousness [9], [10], [11], [12], [13].

Some understanding of FEF interactions with other brain locations has been provided by non-human primate studies revealing that the microstimulation of this area yields selective perceptual modulations for stimuli presented within locations corresponding to the receptive fields of the stimulated neurons, but not outside [14], [15]. Likewise, the non-invasive manipulation of the right FEF activity in the human brain by Transcranial Magnetic Stimulation (TMS) has also shown its ability to modulate neural activity in early visual areas [16], [17] and visual performance on the detection of high-contrast and masked targets [18], [19]. All together those studies suggest that frontal activity has the potential to modulate the processing of visual stimuli, particularly under challenging perceptual conditions. Nonetheless, the processes underlying the ability of this specific cortical frontal site to influence and eventually ameliorate visual perception, particularly when manipulated during the time period preceding the onset of a visual target remain debated.

In the current study, we used single TMS pulses to modulate FEF pre-target activity and studied its impact on the conscious perception of low-contrast near-threshold targets (Experiment 1). Given that neurostimulatory effects have been shown to depend on the pre-existing patterns of activity within the targeted region [20], [21], we then made use of visuo-spatial cues, likely to modulate neural activity along the dorsal attentional orienting network, to study whether the effects of pre-target FEF TMS interacted or not with the state of activity within that network (Experiment 2). The topic holds the potential to provide novel insights on the role of right FEF activity on conscious visual perception and could also help settle the bases in an upcoming near future, for new strategies to manipulate such region with the goal of enhancing human perceptual capabilities.

## Materials and Methods

A group of thirteen participants (8 women and 5 men) aged between 18 and 28 years (average: 24 years old) took part in the study. All participants reported no history of neurological or psychiatric disorders and normal or corrected-to-normal visual acuity. They were all naïve as to TMS and the purpose of the experiments and participated voluntarily. The research protocol and inform consent was reviewed and sponsored by the Inserm (*Institut National de la Santé et la Recherche Scientifique*) ethical committee and approved by an Institutional Review Board (CPP *Ile de France 1, Hôpital de la Pitié-Salpêtrière*). Written informed consent was received from all participants in the study prior to participation. Participants took part in two experiments (Experiment 1 and 2), the order of which was counterbalanced across subjects.

### Apparatus, Visual Stimuli, and Tasks

**Figure 1:**
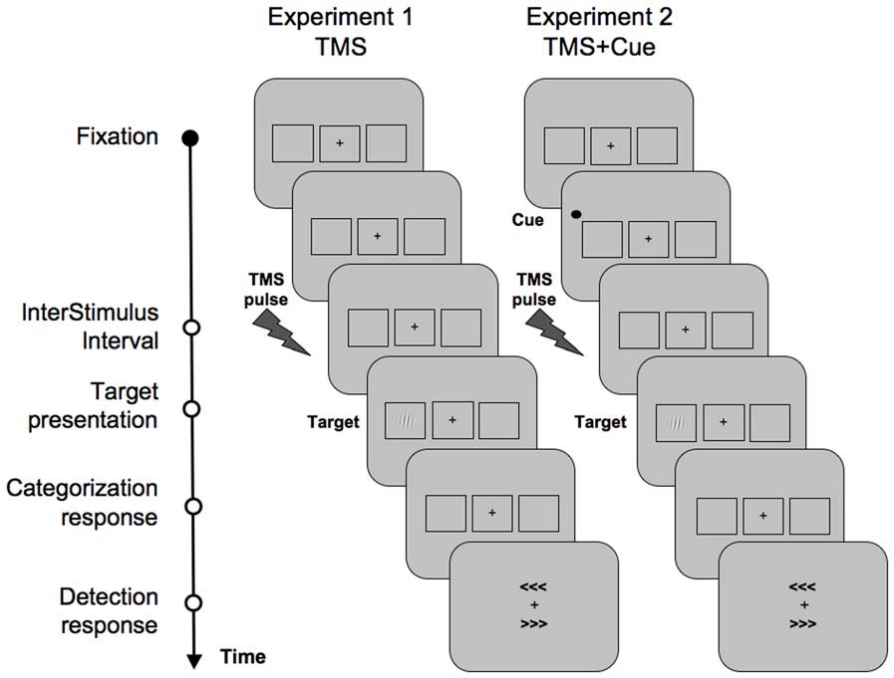
Sequence of events during a representative trial of Experiment 1 (left) and Experiment 2 (right). In both experiments, participants were requested to fixate at a central cross for a randomly variable period of time between 1000 to 1500 ms. In Experiment 1, the fixation cross became slightly bigger for 66 ms and was followed by an active or a sham single TMS pulse delivered on the right FEF, 80,100 or 140 ms prior to target onset. In Experiment 2, a peripheral visuo-spatial cue, consisting in a black circle was displayed for 66 ms to the right or the left of the fixation cross. The cue was predictive about the location of the subsequent target (75% valid and 25% invalid trials), and was followed by a TMS pulse delivered 80 ms pre-target onset. In both experiments active or sham TMS pulses were interleaved in a randomized order. Then, after an interstimulus interval (ISI) of 233 ms, a *Gabor* with the lines tilted to the left or the right appeared for 33 ms at the center of one of the two lateral boxes. Participants were then requested to perform two sequential tasks; first a visual line *categorization* task to indicate the orientation of the *Gabor* lines (left/right) and second, a conscious visual *detection* task in which they had to report if they did see the target, and where they saw it (left/right). A cue is considered valid when it correctly signals the location of the upcoming target (left or right), and invalid when it incorrectly signals target location. A valid trial is the one including a valid cue and the opposite applies to invalid trials. The figure shows for Experiment 2 an example of a valid trial (see Material and Methods for full details on the behavioral paradigms).

Visual stimuli were displayed on an eye tracker screen (Tobii T50, Technology AB, Danderyd, Sweden, 17′′ wide, 1024×768, 16.67 ms refresh rate) using a laptop computer (Dell Latitude E6400, Round Rock, Texas, USA) and standard stimulus presentation software (E-prime, Sharpsburg PA, USA). All stimuli were presented against a grey background (RGB: 194, 194, 194) (Figure 1) and eye movements were controlled throughout each trial. The fixation point (a black “+” sign of 0.5×0.5°) was displayed in the center of the screen, along with three black squared boxes (6.0° width×5.5° height), one central and two lateral ones (centered 8.5° to the left and right of the fixation point). The target consisted of a *Gabor* stimulus (2 cycles/deg. spatial frequency, 3.0° diameter, 0.3° of SD, minimum and maximum Michelson contrast of 0.062 and 0.551, respectively), which could appear at the center of one of two lateral boxes for a brief period of time (33 ms). The lines of the *Gabor* were tilted 1° to 10° to the left or to the right (corresponding 0° to their vertical orientation). Participants were requested to keep fixation on the central cross throughout the trial and to execute two consecutive tasks after the presentation of the target. They were first asked to determine line orientation (*categorization* task), as fast and as accurately as possible, by pressing the corresponding button on a computer keyboard with the index and middle finger of their right hand (“1” for left and “2” for right). In this task, we encouraged them to respond to every trial within a window of 2000 ms, and to guess a response even when the target was not presented or they did not consciously perceive it. Performance was assessed through accuracy and reaction time measures. Secondly, participants were required to report whether they had consciously seen the target or not (*detection* task). To do so, two arrow-like stimuli, one below and one above the fixation cross (>>> and <<<), pointing to the left and to the right side of the screen were presented. Participants were provided with three keys, which they had to operate with their left hand: an upper key (“d”), a lower key (“c”) and the space bar. The upper key signaled the side of the screen pointed by the arrow presented in the upper part of the fixation point, while the lower key was associated to the side of the screen pointed by the lower arrow. Participants had to respond by pressing the space bar if they did not see the stimulus, or, if they did see it, with the corresponding key (“d” or “c”) to indicate the location where the target had been consciously perceived (left or right). The position of the arrows pointing left or right was randomized across trials. Perceptual sensitivity (d’) and response bias (beta) used in Signal Detection Theory [22], [23] served to assess performance in this task. The former (d’) is a bias-free statistic that provides a measure of observers’ ability to detect weak signals, while the latter (beta) describes their relative preference for one response over the other. To compute those two parameters, trials in which the location of a target presented in the screen was correctly determined by participants, were considered as correct detections or “hits”; trials in which the presence of a present target was not acknowledged by participants were considered as “misses”; trials in which participants reported the location for targets that were not presented on the screen were treated as “false alarms”; trials in which the target was absent and participants correctly reported not to have seen it were considered “correct rejections”; and finally, trials in which the location of a present target was incorrectly reported by participants (4% of the ‘seen’ targets in both experiments) were excluded from the analyses as errors.

A titration procedure performed prior to the experimental trials allowed to determine, in each experiment and for each participant, the stimulus contrast at which ∼62% of the displayed targets were consciously reported in the *detection* task and the degree of line tilting for which performance in the *categorization* task remained between 65 and 85% correct. Participants started the titration trials with a high contrast stimulus and, every 20 trials, target contrast and line tilting were adjusted in order to converge to the above-mentioned criteria. Experimental trials started once such performance levels were attained and during the experiment, this whole set of stimulus parameters was also automatically adjusted every 20 trials to avoid behavioral fluctuations caused by task practice or fatigue.

In Experiment 1, every trial started with a fixation screen lasting randomly from 1000 to 1500 ms in order to achieve an inter-trial interval of at least two seconds. The fixation cross became then slightly bigger (0.7×0.7°) for 66 ms to signal the upcoming event. After an Inter Stimulus Interval (ISI) of 233 ms, the target could appear at the center of one of the two lateral boxes. The experiment consisted of 600 trials, including 120 trials in which the target was absent. In half of the trials, chosen randomly, a single TMS pulse was delivered on the right FEF either at 80, 100 or 140 ms prior to the target onset (active TMS trials). In the other half (sham TMS trials), a single pulse was delivered, at those same timings, by a second TMS coil placed next to the stimulation site, with the coil surface perpendicular to the head surface, preventing the magnetic field from reaching the skull and stimulating the brain.

In Experiment 2, everything was kept the same as in Experiment 1 except for the following. The fixation sign did not increase its size but, instead, a visuo-spatial cue, consisting of a black circle (1.5° diameter), was presented in the upper external corner of one of the two lateral boxes and displayed for 66 ms. After the same ISI (233 ms), the target could appear at the center of the cued (valid trial) or uncued (invalid trial) lateral box. The cue was predictive about the location of the upcoming target (75% valid and 25% invalid trials). A cue was considered valid when it correctly signaled the location of the upcoming target (left or right), and invalid when it incorrectly signals target location. A valid trial was the one including a valid cue whereas the opposite applied to invalid trials. Similarly, validly cued targets were those preceded by a valid cue, whereas invalidly cued targets were preceded by an invalid cue. The experiment consisted of 800 trials, including 160 target-absent trials. Active or sham TMS pulses were only delivered 80 ms pre-target onset, given the inability to test all three timings and keep the session within a reasonable duration. Prior experiments suggested that short pre-target timings had the highest potential to induce behavioral effects [19].

### Transcranial Magnetic Stimulation (TMS)

**Figure 2:**
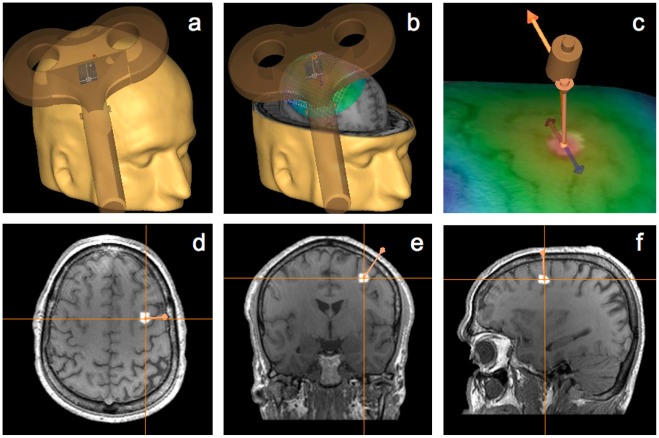
TMS targeted region, neuronavigation and coil placement. The specific location of the right FEF was identified and labeled in a three dimensional reconstruction of each participant’s MRI. The area was targeted with a 70 mm figure-of-eight TMS coil guided by a frameless stereotaxic neuronavigation system (a and b). The active TMS coil was placed flat with its center tangential to the targeted site and oriented lateral to medial and rostral to caudal orientation (c), approximately parallel to the medial portion of the central sulcus, i.e., ∼ a 45° angle with respect to the interhemispheric fissure. See axial (d), coronal (e) and sagittal (d) MRI views of the location for the TMS targeted right FEF (see Material and Methods for full details on the targeting strategy).

TMS pulses were delivered using a biphasic repetitive stimulator (Superapid^2^, Magstim, Withland, UK) with a 70 mm diameter figure-eight air-cooled coil (Figure 2). Pulses were triggered through E-prime software (E-prime, Sharpsburg PA, USA) running on a laptop computer (Dell, Latitude 6410). Prior to the experimental tasks, a structural T1-weighted MRI scan was acquired for every participant at the CENIR MRI center (*Hôpital de la Pitié-Salpêtrière*, Paris). A 3T Siemens MPRAGE sequence, flipangle = 9, Repetition Time = 2300 ms, Echo Time = 4.18 ms, slice thickness = 1 mm, was used. For the TMS experiments, the right FEF region was localized using previously identified Talairach coordinates x = 31, y = −2, z = 47 [24] and labeled with a 0.5 cm radius spherical Region of Interest (ROI) in the MNI space with the Marsbars toolbox (Sourceforge.net). Using SPM5 software (UCL, London, UK), each participant’s structural MRI image was segmented into white and gray matter and the inverse segmentation matrix was used to individually de-normalize the ROI (spatial smooth isotropic Gaussian Kernel of 1-mm full-width half-maximum). The same software was used to co-register the de-normalized ROI with each participant structural MRI volume, obtaining a precise individual localization of the area. The final MRI was uploaded into a frameless stereotaxic system (eXimia NBS System, Nexstim, Helsinki, Finland) and reconstructed in 3D for online neuronavigation of the TMS coil. Given the small size of the region and the high inter-individual variability in FEF location, a TMS-guided individual functional confirmation of the location of the right FEF was conducted by following a well-established protocol based on evidence that a single TMS pulse delivered on the FEF during the preparation time of a saccade has the ability to delay its onset [25].

At all times, the active TMS coil was held tangential to the skull, with its handle oriented ∼45° in a rostral-to-caudal and lateral-to-medial orientation, i.e., parallel to the central sulcus. The TMS coil was kept steady within an area of ∼2 mm radius from the targeted region by using online neuronavigation feedback on each participant’s structural MRI. For all interventions, stimulation intensity was initially set up for every subject at 67% of the TMS machine maximum output. Nonetheless, in some participants, intensity had to be slightly decreased to abolish temporal involuntary muscle activation, involuntary blinks or other types of facial sensations. The average intensity at which participants were stimulated was 66±1% for both experiments (113±12% and 111±15% of the mean resting motor threshold in Experiments 1 and 2, respectively).

### Data Analysis

Trials in which participants showed response anticipations, i.e. pressed the button before stimulus presentation (0.02% and 0.01% of all trials respectively), or broke fixation and performed eye movements to one of the lateral boxes (3% and 6% of all trials for Experiment 1 and 2, respectively) were eliminated from the analyses. The first three participants taking part in Experiment 1 could not be included in the analyses due to a software programming error.

As accuracy in the *categorization* task was high when participants correctly reported to have seen the target (74% in both experiments) and remained at chance levels when they reported not to have seen it (51% and 49% in Experiment 1 and 2, respectively), only correctly seen target trials were considered for reaction time and accuracy analyses. For each timing (80, 100 and 140 ms), TMS condition (active or sham TMS) and validity (valid and invalid), trials with reaction time faster than 150 ms and outside 4 standard deviations of the mean (0.1% and 0.4% in Experiment 1 and 2, respectively) were eliminated from the analyses as outliers.

All behavioral outcomes (accuracy and reaction time for the *categorization* task and perceptual sensitivity and response bias for the *detection* task) were subjected to a repeated measures ANOVA with timing (80, 100 and 140 ms), target location (left and right) and TMS condition (active and sham TMS) as within-participant factors in Experiment 1 and with validity (valid and invalid), target location and TMS condition as within-participant factors in Experiment 2. Such analysis was also performed for detection errors (i.e. target-present trials in which participants incorrectly indicated target location) to rule out any potential TMS effects on these specific types of events. In Experiment 1, no significant main effects or interactions were observed in such trials. In Experiment 2, only a main effect of validity was observed, indicating that participants made fewer errors in valid than invalid trials (F(1, 12) = 13.64, p = 0.003).

## Results

**Figure 3:**
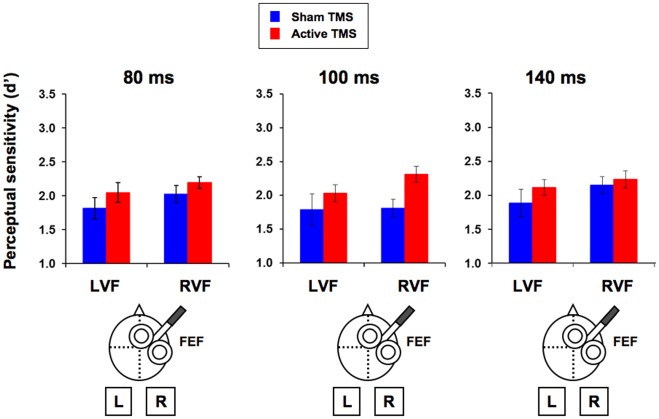
TMS-induced modulations of right FEF pre-target activity on conscious detection (Experiment 1). Perceptual sensitivity (mean ± SE) for the three different timings (80, 100 and 140 ms pre-target onset) used in Experiment 1. Data is presented separately for targets displayed in the visual field contralateral (left visual field, LVF) or ipsilateral (right visual field, RVF) with respect to the targeted right FEF under active (red) or sham (blue) TMS stimulation. A main effect of TMS condition was observed, with higher perceptual sensitivity scores under active than sham TMS pulses (F(1,9) = 8.31, p = 0.018). Based on the a priori hypothesis that such effect depended on timing, we performed three separate repeated measures ANOVA for the three timings. The TMS effect only reached significance when pulses were delivered 80 ms pre-target onset (F(1, 9) = 9.77, p = 0.012), but not when applied 100 ms (F = 5.09, p = 0.051) nor 140 ms (F = 3.95, p = 0.078) pre-target onset.

In Experiment 1, we used single TMS pulses on the right FEF to test the ability of pre-target activity on this region to modulate conscious visual perception of low-contrast near-threshold targets. Participants correctly reported to have seen the target in 56% of the present-target trials and the mean rate of false alarms was 2%. All measures (accuracy and reaction time for the *categorization* task and perceptual sensitivity and response bias for the *detection* task) were subjected to a repeated measures ANOVA with timing (80, 100 and 140 ms), target location (left and right) and TMS condition (active and sham TMS) as within-participant factors. In the *categorization* task, no significant effects of TMS condition were observed. Only a main effect of target location in reaction time reached significance (F(1,9 = 7.88, p = 0.020), participants being faster for targets displayed on the right than on the left visual hemifield. Responses also proved to be more accurate when responding to right than left targets (F(1,9) = 6.68, p = 0.030). In contrast, in the *detection* task, a main effect of TMS condition reached significance, with overall higher perceptual sensitivity (d’) under active than sham TMS pulses (F(1,9) = 8.31, p = 0.018). On the basis of the a *priori* hypothesis that stimulation should depend on pulse delivery time, we performed three separate repeated measures ANOVA for the three TMS timings, with side and TMS condition as within-participant factors. The TMS effect only reached significance when pulses were delivered 80 ms pre-target onset (F(1, 9) = 9.77, p = 0.012), but not when applied 100 ms (F = 5.09, p = 0.051) or 140 ms (F = 3.95, p = 0.078) pre-target onset (Figure 3 and Table 1). No main effects or interactions reached statistical significance for the response bias (beta).

**Table 1:** Data from TMS-induced modulations of right FEF pre-target activity on visual performance (Experiment 1).

Task	Mean values±SE	TMScondition	80 ms			100 ms		140 ms			
			LVF	RVF	LVF		RVF		LVF	RVF	
**Detection**	**d’ score**	**Sham**	1.82±0.16	2.03±0.13	1.79±0.23		1.81±0.13		1.89±0.20	2.15±0.12	
		**Active**	2.05±0.15	2.20±0.09	2.03±0.13		2.31±0.12		2.12±0.11	2.23±0.12	
	**Beta measure**	**Sham**	5.69±0.45	5.66±0.29	4.66±0.56		4.93±0.53		5.38±0.45	5.42±0.35	
		**Active**	5.60±0.37	6.84±0.34	5.91±0.33		5.50±0.37		5.90±0.31	5.32±0.43	
**Categorization**	**RT (ms)**	**Sham**	849±55	778±51	805±38		767±49		814±49	779±58	
		**Active**	840±52	792±53	833±52		776±41		834±50	789±40	
	**Accuracy**	**Sham**	0.68±0.03	0.79±0.04	0.70±0.04		0.78±0.03		0.76±0.04	0.72±0.03	
		**Active**	0.77±0.02	0.79±0.02	0.69±0.03		0.76±0.02		0.69±0.04	0.75±0.02	

Perceptual sensitivity (d’ scores, mean ± SE) and response criterion (beta measures, mean ± SE), and reaction time (RT) (mean ± SE) and accuracy (mean ± SE), for the three different TMS delivery timings (80, 100 and 140 ms pre-target onset), obtained respectively for the conscious visual *detection* and visual *categorization* tasks explored in Experiment 1. Data are presented for targets displayed in the visual field contralateral (left visual field, LVF) and ipsilateral (right visual field, RVF) with respect to the stimulated right FEF under the effects of active or sham TMS pulses.

In Experiment 2, FEF TMS was delivered after the engagement of the dorsal attentional orienting network by a peripheral visuo-spatial cue, which was predictive about the location of the subsequent target. Given our purpose of studying the combined effects of a single TMS pulse and a cue-driven engagement of attentional orienting, only participants effectively orienting their attention according to the cue, and thus exhibiting cueing effects under sham TMS trials, were considered for further analyses. An assessment of the perceptual effects induced by visuo-spatial attentional orienting using the exact same paradigm (see Experiment 4 in [26] for details) demonstrated that, for this very same *categorization* task, effective visuo-spatial attentional orienting entailed significant reaction time reductions in valid as compared to invalidly cued targets. Accordingly, the presence of a significant cueing effect was statistically assessed in our participants by comparing the mean reaction time of valid vs. invalid sham TMS trials. Seven out of the thirteen participants showed statistically significant reductions of reaction time for valid vs. invalid sham TMS trials (unpaired 1 tailed t-test, p<0.05) and thus were considered as exhibiting cueing effects.

**Figure 4:**
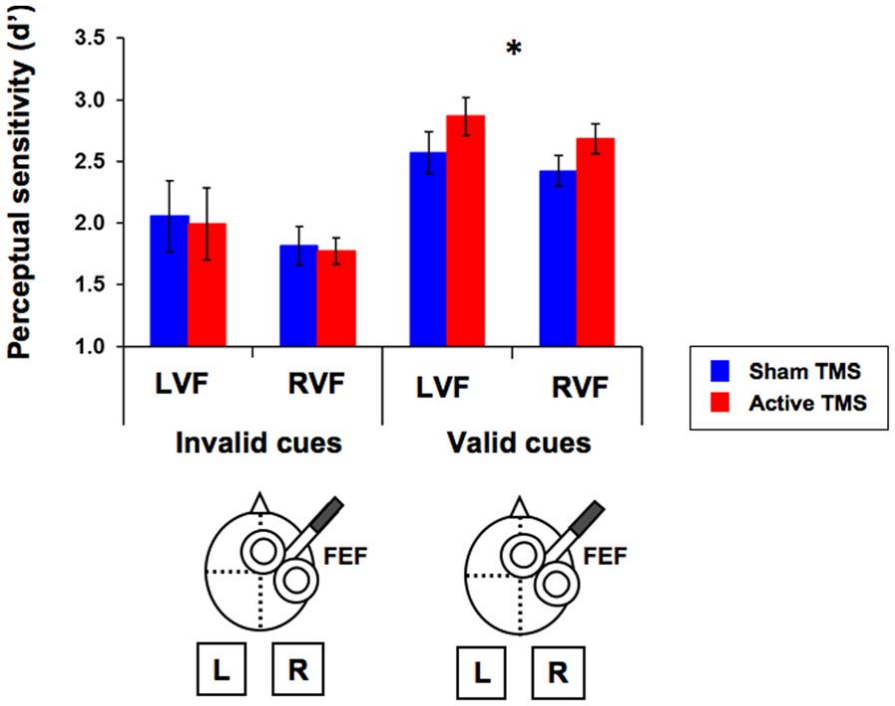
TMS-induced modulations of FEF pre-target activity on conscious detection after cue-driven attentional orienting (Experiment 2). Perceptual sensitivity (mean ± SE) for targets displayed in the visual field contralateral (left visual field, LVF) or ipsilateral (right visual field, RVF) with respect to the stimulated right FEF site under active TMS (red) or sham TMS (blue). An interaction between validity and TMS proved statistically significant (F(1, 6) = 6.54, p = 0.043) indicating that, when delivered after the presentation of a peripheral predictive visuo-spatial cue, TMS pulses yielded significant bilateral enhancements of conscious visual detection only when the cue correctly signaled the location of the subsequent target (valid trials, F = 19.26, p = 0.005, indicated by the asterisk), whereas no effects were observed when the cue incorrectly predicted it (invalid trials, F<1).

Those participants reported to have seen the target in 58% of the present-target trials and the mean rate of false alarms was 6%. All measures (accuracy and reaction time for the *categorization* task and perceptual sensitivity (d’) and response bias (beta) for the *detection* task) were subjected to a repeated measures ANOVA with validity (valid and invalid), target location (left and right) and TMS condition (active and sham TMS) as within-participant factors. In the *categorization* task, only a main effect of validity in reaction time reached significance (F(1, 6) = 60.22, p<0.001), with faster responses for valid than invalid trials. In the *detection* task, a significant interaction between validity and TMS condition was observed on perceptual sensitivity (F(1, 6) = 6.54, p = 0.043), indicating the dependency of TMS effects on the validity of the cue. More specifically, active stimulation improved perceptual sensitivity (d’) only when the cue correctly predicted the location of the target (valid trials), as compared to sham TMS (F = 19.26, p = 0.005). Interestingly, no differences between active and sham TMS were observed for invalid trials, in which the cue incorrectly predicted the location of the target (F<1) (Figure 4, Table 2). No significant main effects or interactions were observed for the response bias (beta).

**Table 2:** Data from TMS-induced modulations of FEF pre-target activity on visual performance after cue-driven attentional orienting (Experiment 2).

Task	Meanvalues±SE	TMScondition	Invalid		Valid	
			LVF	RVF	LVF	RVF
**Detection**	**d’ score**	**Sham**	2.05±0.77	1.81±0.41	2.57±0.45	2.42±0.33
		**Active**	1.99±0.78	1.77±0.28	2.87±0.40	2.68±0.32
	**Beta Measure**	**Sham**	15.67±6.57	15.73±4.87	9.73±6.89	13.41±6.54
		**Active**	13.49±5.21	14.94±4.76	10.44±4.79	14.09±7.13
**Categorization**	**RT (ms)**	**Sham**	910±175	825±74	717±133	730±105
		**Active**	832±136	820±120	719±128	719±97
	**Accuracy**	**Sham**	0.73±0.18	0.79±0.03	0.73±0.05	0.79±0.04
		**Active**	0.79±0.14	0.83±0.10	0.74±0.05	0.74±0.06

Perceptual sensitivity (d’ scores, mean ± SE) and response criterion (beta measures, mean ± SE), and reaction time (RT) (mean ± SE) and accuracy (mean ± SE), for the conscious visual *detection* and visual *categorization* tasks explored in Experiment 2. Data are presented for valid and invalid trials, in which targets were displayed in the visual field contralateral (left visual field, LVF) and ipsilateral (right visual field, RVF) to the stimulation site (right FEF), under the effects of active or sham TMS pulses.

## Discussion

The potential of non-invasive brain neurostimulation to boost cognitive performance beyond the limits set up by individual skills and capabilities in healthy [27], [28], [29], [30], [31], [32], [33] and pathological states [34], [35], [36], [37], [38] has been postulated for more than a decade. Thanks to its ability to activate discrete cortical regions and associated networks [39], TMS, a focal magnetically-based non-invasive brain stimulation technique, has been shown to induce punctual or lasting changes in the firing patterns of restricted key cortical regions and, in virtue of such capabilities, influence normal or pathological human behavior [40], [41]. We hereby assayed in healthy humans whether conscious visual perception of low-contrast near-threshold targets could be enhanced with non-invasive neurostimulation, by modulating the activity of the right FEF prior to the onset of a visual target. Such brain region has been shown to be involved in visuo-spatial attentional orienting [7] and also to have bearing on conscious access [9], [10], [11], [12], [13] for visual stimuli. In agreement with prior work [14], [15], [18], [19], [42], our data from Experiment 1 indicate that right FEF pre-target activity is indeed relevant for conscious perception and that its non-invasive manipulation with TMS can induce relevant visual perceptual sensitivity improvements. Interestingly, when the dorsal attentional orienting network was previously activated by means of peripheral predictive visuo-spatial cues (Experiment 2), the modulation of right FEF pre-target activity with TMS pulses brought visual perceptual sensitivity modulations, which were shaped according to cue validity. More specifically, only when the prior visuo-spatial cue correctly predicted the site (left or right) of the subsequent target (valid trials) but not when it incorrectly predicted it (invalid trials), TMS induced facilitatory effects on conscious detection. These results suggest that cue-driven neural activations related to attentional orienting interact with conscious perception and have the potential to sculpt the effects of time locked pre-target FEF stimulation and render such perceptual facilitatory outcomes more specific. In spite of the lack of an active control condition mimicking not only the TMS clicking noise but also the scalp tapping sensations, the lack of significant effects when TMS pulses were combined with invalid spatial cues became an internal control that rules out a hypothetical contribution of such phenomena to our results.

Prior studies have demonstrated that the impact of non-invasive neurostimulation can be highly influenced by the pre-existing patterns of activity within the stimulated region and its associated networks [20], [21]. In our experiments, visuo-spatial cues could have differentially modulated the firing patterns of distinct neuronal subpopulations within the right FEF region, prior to the onset of neurostimulation, and hence have primed the effects of FEF TMS only for those under certain states of activation. In support of this hypothesis, non-human primate research has shown that peripheral predictive visuo-spatial cues increase (and maintain increased along the cue-to-target period) the firing patterns of the FEF neurons that specifically code for the signaled location, but not for those whose receptive fields lay outside the cued site [43]. On the basis of this observation, different activity levels or ‘states’ of activation across FEF neuronal subpopulations as driven by visuo-spatial cues could easily explain how, on a trial-by-trial basis, highly selective visual facilitation patterns could emerge from the stimulation of roughly the same cortical resources as a function of cue validity [44].

Our data indicate that the FEF TMS visual facilitatory effects interacted with the orienting of spatial attention engaged by means of predictive spatial cues. Nonetheless, given the frequently hypothesized role of the right FEF not only as a crucial node of the dorsal attentional network but also as relevant in providing access to consciousness, which of these two systems might have been ultimately responsible for the observed visual facilitatory effects remains unclear. Contributing to the discussion of this issue, our data reveal that FEF TMS neither when used in isolation (Experiment 1) nor when combined with visuo-spatial cues (Experiment 2) did modulate the reaction times or accuracy levels for the visual *categorization* task. A behavioral study performed and published separately by our group assessed the behavioral effects of visuo-spatial attentional orienting in the same exact paradigm, and showed significant shorter reaction times in response to stimuli presented at attended than unattended locations (see [26] Experiment 4 for details). The latter effects, which were accompanied by a modulation in perceptual sensitivity in the *detection* task only when the cue was predictive about target location, strongly suggest that cue-validity effects in such paradigm should be considered a solid signature of attentional orienting. On such basis, it is tempting to interpret the current lack of reaction time modulations for the *categorization* task, accompanying improvements in visual *detection* by FEF pre-target activity modulations, not as ultimately mediated by the manipulation of visuo-spatial orienting processes but reflecting a genuine effect of right FEF TMS on visual consciousness. In spite of obvious differences between intact and damaged systems, this interpretation could be in agreement with patient work showing a relevant role of the prefrontal cortex in access to consciousness of masked stimuli, not accountable either by attentional orienting processes [45]. Nonetheless, given that attention can alter appearance [3] and that in our paradigm composed of two serial tasks, subjects could have eventually sacrificed reaction time for accuracy, or categorization performance for detection performance, whether attention can modulate conscious visibility without affecting reaction time remains an open question.

Our data contribute further evidence in support of the notion that the right FEF and its associated systems may constitute according to monkey [14], [15], [42] and human [19], [44] data, a key area facilitating access to consciousness for visual stimuli. Moreover, our combined modulation strategy based on an ‘at will’ stimulation of the FEF and the presentation of visuo-spatial cues, showed its ability to selectively enhance human visual awareness for low-contrast near-threshold stimuli and to shape the specificity of such effects, thus setting up the stage for the use of TMS on the direct manipulation of visual conscious perception in healthy and pathological states. Unfortunately, in absence of brain neuroimaging data, we cannot yet rule out if such facilitatory phenomena were driven locally at the stimulated right FEF and directly manipulated by the alleged ability of this area to contribute to visual awareness; emerged from connectivity-conveyed trans-synaptic effects on primary visual regions through fronto-parietal-occipital top-down projections [17], [46]; or resulted from the modulation of other intermediate cortical or subcortical structures interconnected with the FEF. This remains a highly relevant question to be addressed in an immediate future through specific experiments which, as elegantly performed elsewhere [17], [46] might require the combination of stimulation and neuroimaging. Moreover, in the current study, we manipulated activity patterns within the right FEF since this area is a key component of the dorsal network devoted to visuo-spatial attentional orienting; its anatomical location can be individually confirmed through a well-established mapping procedure; there is precedence on its ability to induce connectivity mediated functional modulation on visual regions, and in consideration of its hypothesized role in visual awareness. In agreement with findings suggesting the dominant role of the right hemisphere sites in attentional orienting and consciousness [18], [19], [27], our intervention in the right FEF proved similarly efficacious for right and left targets. Prior studies have also reported bilateral effects for right FEF activity modulations, whereas the manipulation of the left FEF stimulation would be restricted to an influence on targets presented in the right visual hemifield. Future venues will have to explore the role of left FEF pre-target activity in conscious visual perception and the extent of such effects throughout the visual field. Furthermore, functional MRI and TMS brain-function studies suggest that the modulation of non necessarily frontal regions, such as the right intraparietal sulcus or the angular gyrus [7] could potentially also interact with cue validity and result in visual facilitatory effects, and thus they would also deserve to be explored in similar paradigms in the future.

In sum, our findings show that FEF pre-target activity can be effectively manipulated to influence conscious visual perception using non-invasive neurostimulation methods, and that a combined strategy based on right hemisphere frontal stimulation and visual cues can be implemented not only to episodically enhance visual performance, but to shape the selectivity of those effects. The fact that a combination of TMS and attentional cues can indeed improve visual sensitivity should be considered a proof of concept that visual capabilities can be manipulated and improved through those approaches. On that basis, strategies operating on cerebral sites involved in attentional orienting and conscious access could become a reality to punctually increase visual capabilities in healthy participants. Similar principles could be also applied to clinical rehabilitation, aiming at containing visual acuity losses in patients with retinal defects, and allowing the emergence of episodic or lasting periods of conscious vision in cortically damaged patients. Nonetheless, it should also be strongly emphasized that the ameliorations demonstrated in our study operate trial-by-trial and remain extremely short lasting. Furthermore they have been demonstrated for lateralized right or left peripheral detections and thus might not equally occur for targets presented in other locations of the visual hemifield. Both aspects weaken the current applicability of the results for meaningful behavioral ameliorations in healthy individuals or therapeutic applications in patients. In order to overcome such limitations, however, longer rTMS patterns and multi-day rTMS regimes combined with spatial cuing paradigms remain to be studied and evaluated for their ability to generate lasting increases in visual sensitivity. Similarly, the differential ability of TMS based approaches to generate ameliorations for targets presented at different visual field locations than those tested in the current paper would need to be studied before our findings could be considered potentially interesting for clinical applications.
